# Polymorphisms in lncRNA PTENP1 and the Risk of Gastric Cancer in a Chinese Population

**DOI:** 10.1155/2017/6807452

**Published:** 2017-08-28

**Authors:** Yugang Ge, Yu He, Mingkun Jiang, Dakui Luo, Xiangkun Huan, Weizhi Wang, Diancai Zhang, Li Yang, Jundong Zhou

**Affiliations:** ^1^Department of General Surgery, The First Affiliated Hospital of Nanjing Medical University, Nanjing, China; ^2^Suzhou Cancer Center Core Laboratory, Nanjing Medical University Affiliated Suzhou Hospital, Suzhou, Jiangsu, China

## Abstract

Long noncoding RNA (lncRNA) phosphatase and tensin homolog pseudogene 1 (PTENP1) is significantly downregulated in gastric cancer (GC), playing critical roles in GC progression. However, the association between PTENP1 genetic variants and GC risk has not yet been reported. Using TaqMan technology, three lncRNA PTENP1 tag single nucleotide polymorphisms (tagSNPs) (rs7853346 C>G, rs865005 C>T, and rs10971638 G>A) were genotyped in 768 GC patients and 768 cancer-free controls in a Chinese population. We found that subjects with rs7853346 G allele had a remarkably decreased risk of GC, compared with those carrying C allele (*P* = 0.011 in an additive model, *P* = 0.033 after Bonferroni's correction). The further stratified analyses showed that the link between variant genotypes of rs7853346 and decreased GC risk was more obvious in older subjects (≥60 years), nonsmokers, nondrinkers, and subjects without family history of GC. We also found that relative PTENP1 mRNA expression levels were higher in rs7853346 CG/GG genotype carriers than those with common genotype in both GC and normal tissues (*P* < 0.05). Besides, bioinformatics analyses revealed that rs7853346 may change the local folding structure and alter the target microRNAs (miRNAs) of PTENP1. In conclusion, our results suggested that lncRNA PTENP1 polymorphism rs7853346 may predict GC susceptibility.

## 1. Introduction

Gastric cancer (GC) is one of the most common cancers worldwide, whose incidence rate is higher in Eastern Asia generally (particularly in Korea, Mongolia, Japan, and China) [1]. The National Central Cancer Registry of China (NCCR) demonstrated that both incidence and mortality of GC rank the second among all cancers in China [2]. The occurrence and development of GC are influenced by a series of complex factors involving host genetic susceptibility and multiple environmental factors [3]. In previous epidemiological studies, several genetic polymorphisms were identified to impact the risk of GC [4–7].

Long noncoding RNAs (lncRNAs) are defined as transcripts more than 200 nucleotides in length, which are associated with a variety of biological progress through interacting with DNA, RNA, and proteins as molecular scaffolds, sponges, or activators [8]. LncRNA PTENP1 is the pseudogene of the phosphatase and tensin homolog (PTEN), sharing high sequence homology with PTEN. Recently, accumulating evidences revealed that PTENP1 was downregulated or deleted in various cancers, such as GC [9], hepatocellular carcinoma (HCC) [10], renal cell carcinoma [11], head and neck squamous cell carcinoma (HNSCC) [12], melanoma [13], endometrial cancer [14], and oral squamous cell carcinoma (OSCC) [15]. In GC, Zhang et al. [9] found that PTENP1 served as a competing endogenous RNA (ceRNA), which decoyed a series of miRNAs (miR-106b, miR-93) binding to 3′ UTR of PTEN and modulated the expression of PTEN. PTENP1 was decreased in GC samples and its overexpression inhibited cell growth, migration, and invasion as well as induced apoptosis. Another previous study illuminated that lower expression of PTENP1 in GC tissues or cell lines could be partly correlated with DNA hypermethylation, and it was related to larger tumor size, more advanced TNM staging, deeper invasion depth, and lymph node metastasis [16]. Furthermore, Dong et al. [17] gave evidence that PTENP1 was also downregulated in GC patients sera, combined with CUDR, LSINCT-5, which could predict clinical outcomes of patients. The current studies about GC mainly focused on the effects of lncRNA PTENP1 on tumor progression, not covering gastric carcinogenesis. To our knowledge, no relevant studies were conducted to investigate the relationship between genetic variants of PTENP1 and the risk of malignant diseases, including GC, in a Chinese population or other populations.

Considering the role of PTENP1 in GC suppression, based on the 1000 Genomes data, we selected three tagSNPs (rs7853346, rs865005, and rs10971638) in lncRNA PTENP1. The genotyping assay was carried out to evaluate the influence of three tagSNPs on the risk of GC in this hospital-based, case-control study in a Chinese population.

## 2. Materials and Methods

### 2.1. Study Subjects

The case-control study received approval from the ethics committee of the First Affiliated Hospital of Nanjing Medical University. Written informed consents were provided by all participants. All subjects consisting of 768 GC patients and 768 cancer-free controls were consecutively recruited from the First Affiliated Hospital of Nanjing Medical University during May 2010 to December 2016. Besides, there was a portion of nonresponse patients accounting for 10.2 percent of all GC patients. The participants, who lived in Jiangsu province or surrounding areas, were genetically unrelated ethnic Han Chinese, and none received blood transfusion from nonself in the preceding six months. All cases were primary and histopathologically confirmed GC by two pathologists, and patients who had any history of malignancies (including any metastatic cancer) and received radiotherapy or chemotherapy previously were eliminated from this study. All controls were randomly selected from vascular surgery or orthopedics department of the abovementioned hospital during the same period, none had self-reported history of malignancies or gastric polyp. We collected relevant information, such as age, gender, hypertension, diabetes, smoking history, drinking history, residence, family history of GC (FH), and clinical data from the medical history, or face-to-face interviews were conducted to supplement the above information. The definitions of hypertension and diabetes were described in the previous studies [5, 6]. The smokers were defined as individuals who formerly or currently smoked ≥10 cigarettes per day no less than 2 years. A drinker was defined as an individual who consumed alcohol at least once a week for more than 1 year [18]. The residence was determined according to the addresses of the participants' electronic medical records. After that, both GC patients and cancer-free controls donated 5 ml peripheral venous blood for DNA extraction and genotyping analysis.

GC tissues and corresponding adjacent tissues were obtained from a total of 70 patients, who underwent surgery at the First Affiliated Hospital of Nanjing Medical University during 2010 to 2014. The resected samples then were frozen with liquid nitrogen immediately and stored at −80°C for RNA extraction. The diagnosis of all patients was histopathologically confirmed by two pathologists.

### 2.2. SNP Selection

Based on Chinese Han in Beijing population data from the 1000 Genomes Project (Ensembl GRCh37 release 87—Dec 2016), we selected tagSNPs of PTENP1 in chromosome 9, position 33673502-33677497 (http://asia.ensembl.org/index.html). The pairwise option of Haploview version 4.2 software (Cambridge, MA, USA), setting *r*^2^ > 0.8 [19], was performed to single out tagSNPs with a minor allele frequency > 0.05. Finally, three tagSNPs (rs7853346 C>G, rs865005 C>T, and rs10971638 G>A) were selected to capture the common SNPs of PTENP1, which were in accordance with the analysis results of Genome Variation Server 144 (http://gvs.gs.washington.edu/GVS144/).

### 2.3. DNA Extraction and Genotyping

Genomic DNA was extracted from the leukocytes of 2 ml peripheral blood of the subjects using RelaxGene Blood DNA System kit (Tiangen Biotech, Beijing, China), following the manufacturer's directions. The NanoDrop Spectrophotometer (ND2000) was conducted to measure the concentration and purity of all DNA specimens, which were diluted to a concentration of 10 ng/*μ*l and stored at −20°C before genotyping. The genotypes of these three tagSNPs were determined by TaqMan allelic discrimination methods with a 96-well ABI StepOnePlus real-time PCR system (Applied Biosystems Foster City, CA, USA). The 10 *μ*l PCR reaction volume included 1 *μ*l DNA template, 5 *μ*l 2× HotTaq PCR Reaction Mix, 0.5 *μ*l primer, 0.25 *μ*l probe, and 2.5 *μ*l double distilled water. The sequences of the probes and primers were showed in Supplementary Table S1 available online at https://doi.org/10.1155/2017/6807452 (BioSteed BioTechnologies, Nanjing, China). Amplification conditions were as follows: 95°C for 10 min, followed by 40 cycles of 95°C for 15 s and 60°C for 1 min. The call rates were more than 98% for all SNPs. Over 10% of samples were selected randomly for repeated assays, and the results were in line with the previous genotypes.

### 2.4. Real-Time Reverse Transcription PCR Analyses of lncRNA PTENP1

Total RNA was extracted from 70 paired GC and adjacent normal tissues using TRIzol reagent (Invitrogen, Carlsbad, CA, USA), which then was reverse transcribed into cDNA with Primescript RT Reagent (Takara, Japan). Real-time reverse transcription PCR (RT-PCR) was conducted to evaluate relative PTENP1 expression with ABI StepOnePlus real-time PCR system (Applied Biosystems Foster City, CA, USA). All procedures were performed in triplicate. The PTENP1 primers were as follows: forward 5′-TCAGAACATGGCATACACCAA-3′; reverse 5′-TGATGACGTCCGATTTTTCA-3′. *β*-Actin (forward 5′-TCCTCTCCCAAGTCCACACA-3′; reverse 5′-GCACGAAGGCTCATCATTCA-3′) was selected as the endogenous control to standardize data. The 10 *μ*l amplification reaction volume contained 5 *μ*l SYBR Green Master Mix (Vazyme, Nanjing, China), 0.2 *μ*l primer, and 100 ng cDNA. RT-PCR was carried out with the condition: 95°C for 5 min, followed by 40 cycles of 95°C for 10 s and 60°C for 30 s.

### 2.5. Statistical Analysis

The SPSS 22.0 software (SPSS Inc., Chicago, IL, USA) was used for statistical analyses. All *P* values were two-sided, and the data were considered to be statistically significant when *P* < 0.05. The goodness-of-*χ*^2^ test was conducted to assess the Hardy-Weinberg equilibrium among cancer-free subjects. Differences in demographic data between cases and controls were calculated by the *χ*^2^ tests (for categorical variables) or Student's *t*-tests (for continuous variables). The risk caused by genetic variants was assessed with odds ratios (OR) and 95% confidence intervals (CI). Crude ORs were computed by the unconditional univariate analysis and adjusted OR, was evaluated by unconditional logistic regression method with age, gender, smoking condition, drinking condition, hypertension, diabetes, residence, and FH adjusted. The results were corrected with the Bonferroni method conservatively for multiple comparisons in association analyses. The stratification analysis was performed under the dominant model, due to the small sample size of GG genotype group for rs7853346. We carried out the stratified analysis to exclude impacts of the potential confounders on GC risk in the logistic regression model. Moreover, we can concretely assess the magnitude of the interested association in every two strata for all subgroups and calculate the heterogeneity to know whether rs7853346 exists interaction with these stratified factors. The *χ*^2^-based *Q*-test and *I*^2^ by Stata 14.0 software were used to detect the heterogeneity from corresponding subgroups [20, 21]. The relative expression levels of PTENP1 in all samples were calculated with 2^−ΔCT^ method normalized to the levels of *β*-actin. The one-way ANOVA test was adopted to evaluate the associations between the expression levels of PTENP1 mRNA and PTENP1 polymorphisms. The Power and Sample Size Calculation software (PS, version 3.1.2, 2014, http://biostat.mc.vanderbilt.edu/twiki/bin/view/Main/PowerSampleSize) was used to calculate the statistical power.

## 3. Results

### 3.1. Demographic Characteristics

The characteristics of 768 GC patients and 768 cancer-free controls were detailed in Table 1. No remarkable differences were observed between the two groups in the distributions of age, gender, hypertension, diabetes, residence, and FH (*P* > 0.05). However, the percentage of smokers was apparently higher among GC group compared with controls (27.6% versus 18.6%, *P* < 0.001), so was that of drinkers (22.8% versus 12.9%, *P* < 0.001). The Supplementary Table S2 summarized the characteristics of 87 nonresponse GC patients. Compared with included 768 GC cases, the proportion of rural population in nonresponse GC patients was larger (70.1% versus 57.3%, *P* = 0.021).

### 3.2. Associations of Selected SNPs in PTENP1 and GC Risk

The genotype and allele frequencies of PTENP1 tagSNPs in cases and controls as well as their associations with GC risk were calculated by the following five methods (additive, codominant, dominant, recessive models, and allele contrast) (Table 2 and Supplementary Table S3). There were no significant deviations from the Hardy-Weinberg equilibrium for the three SNPs among the controls (*P* = 0.900 for rs7853346, *P* = 0.098 for rs865005, and *P* = 0.342 for rs10971638). For rs7853346, the frequency of G allele of GC cases was significantly lower than that of the controls (*P* = 0.007, OR = 0.79, 95% CI = 0.66–0.94). In the additive model, logistic regression analysis indicated that rs7853346 variant genotypes significantly decreased GC risk (*P* = 0.011, adjusted OR = 0.80, 95% CI = 0.67–0.95). In codominant and dominant models, the variant CG and CG+GG genotypes were significantly associated with the decreased risk of GC(*P* = 0.033, adjusted OR = 0.79, 95% CI = 0.63–0.98 for CG; *P* = 0.015, adjusted OR = 0.77, 95% CI = 0.62–0.95 for CG+GG). After Bonferroni's multiple adjustment applied to our results, we found that the variant genotypes of rs7853346 were still obviously correlated to decreased GC risk in additive and dominant models (*P* = 0.033 and 0.045, resp.). With the increasing number of G alleles, the proportion of controls increased (*P* trend = 0.013, *P* trend = 0.039 after Bonferroni's correction). Based on the fact that the tumor differentiation of 78% GC patients was poor, we took the well + moderate differentiation group as a sensitivity analysis to evaluate the association between rs7853346 and GC risk. As shown in Supplementary Table S4, no impacts of rs7853346 variant genotypes on the susceptibility to GC risk were observed. Using the PS software, given PTENP1 rs7853346 C>G mutant alleles in the control group, OR, GC samples, and control sample sizes, the power of our analysis (*α* = 0.017) was 0.708 in 768 GC cases and 768 controls with unadjusted OR = 0.69.

However, no significant associations were detected between the genotypes of the other two PTENP1 tagSNPs (rs865005 C>T, rs10971638 G>A) and GC risk (*P* > 0.05).

### 3.3. Stratified Analysis of rs7853346 in PTENP1

To eliminate influences of the potential confounders on GC risk, the stratified analysis was conducted for rs7853346 according to age (60 years), gender, smoking status, drinking status, residence, and FH. All results were obtained under the dominant model and summarized in Table 3. We observed a significantly decreased risk of GC in terms of older subjects (age ≥ 60) (*P* = 0.026, adjusted OR = 0.72, 95% CI = 0.54–0.96), nonsmokers (*P* = 0.037, adjusted OR = 0.78, 95% CI = 0.61–0.99), nondrinkers (*P* = 0.016, adjusted OR = 0.75, 95% CI = 0.60–0.95), and non-FH (*P* = 0.023, adjusted OR = 0.78, 95% CI = 0.62–0.97). However, no significant association was identified between rs7853346 and GC risk in terms of sex or residence. The differences in every two strata of all stratified factors were not statistically significant (*I*^2^ = 0%, *P* > 0.05), which showed that these stratified factors (age, gender, etc.) had no multiplicative interaction with rs7853346.

Next, the subgroup analysis was conducted to evaluate the correlations between the variant genotypes of rs7853346 and clinicopathological features in GC patients (Table 4). We found that rs7853346 CG/GG genotypes could decrease the susceptibility to GC in subjects with tumor well + moderate differentiation (*P* = 0.048, adjusted OR = 0.70, 95% CI = 0.49–1.00). Nonetheless, no distinct association was found between rs7853346 and other clinicopathological characteristics, such as tumor infiltration depth, lymph node metastasis, and localization.

### 3.4. Effects of rs7853346 on PTENP1 Secondary Structures and Target miRNAs

The RNAfold algorithm was performed to analyze the impact of rs7853346 on the local secondary structure of PTENP1. As shown in Supplementary Figure S1, the folding structure of PTENP1 was notably changed with rs7853346 C/G alleles.

Given the fact that rs7853346 C>G is located in the 3′ untranslated region (UTR) of PTENP1 gene, we applied the RegRNA2.0 program to predict that rs7853346 C>G may create hsa-miR-1245b-3p, hsa-miR-4679 binding sites on PTENP1.

### 3.5. Functional Relevance of rs7853346 to PTENP1 Expression

**Figure 1:**
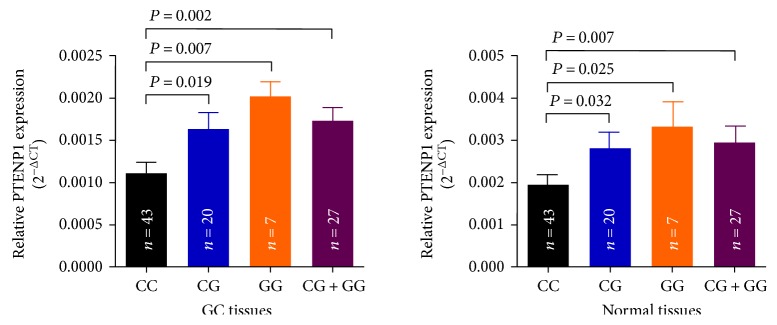
Correlation between rs7853346 genotypes and expression of PTENP1 mRNA. PTENP1 levels were determined by qRT-PCR in GC tissues and adjacent normal tissues in subjects with the CC (*n* = 43), CG (*n* = 20) or GG (*n* = 7) genotype at rs7853346. Results were shown as mean ± standard error relative to *β*-actin levels. The significant level was corrected with the formula of *α*′ = *α*/3 = 0.017 according to the Bonferroni method.

We then evaluated the effects of the rs7853346 genotypes on PTENP1 levels by qRT-PCR in 70 paired GC and adjacent normal samples including 43 CC, 20 CG, and 7 GG genotypes. The results showed that the relative PTENP1 mRNA expression levels (mean ± standard error) were significantly higher for the CG, GG, or CG+GG genotypes than for the CC genotype in both GC and adjacent normal tissues (GC tissues: *P* = 0.019, 0.007, and 0.002, resp.; adjacent normal tissues: *P* = 0.032, 0.025, and 0.007, resp.) (Figure 1). After Bonferroni's correction, GC tissues with GG or CG+GG genotypes had higher expression levels of PTENP1 than CC genotype (corrected *P* = 0.021 and 0.006). In adjacent normal tissues, only the PTENP1 expression levels of those with CG+GG genotypes were higher than CC genotype (corrected *P* = 0.021).

## 4. Discussion

In the case-control study, the associations between lncRNA PTENP1 polymorphisms (rs7853346, rs865005, and rs10971638) and the risk of GC were firstly evaluated in Chinese Han population. Our results showed that variant genotypes (CG/GG) of rs7853346 were associated with a prominent reduced risk of GC. Moreover, PTENP1 expression levels in individuals carrying rs7853346 CG/GG genotypes were higher than CC genotype in both GC and adjacent normal tissues. The statistical power calculated by using the PS Software was 0.708 and was relatively small.

LncRNAs are a class of noncoding RNAs, and increasing evidences have demonstrated that aberrant expression of lncRNAs could result in the occurrence and development of various malignancies [22, 23]. For instance, lncRNA PTENP1, a pseudogene of PTEN, was verified to function as a tumor suppressor in some cancer types, including GC. Liu et al. [12] identified that the downregulation of PTENP1 promoted cell proliferation, colony formation, and migration in HNSCC, and it was also related to poor survival of patients. For GC, Zhang et al. [9] reported that PTENP1 functioned as a ceRNA to regulate PTEN level by sponging miR-106b and miR-93 in GC. Similarly, the overexpressed PTENP1, sequestering miR-17, miR-19b, and miR-20a, upregulated PTEN, PHLPP, and such autophagy genes as ATG7, ULK1, and p62, which in turn repressed the tumorigenic properties of HCC cells [10]. However, as far as we know, no related reports have revealed whether PTENP1 polymorphisms have influence on the susceptibility to GC in a Chinese population.

Subsequently, our stratified analysis by age showed that the decreased GC risk related to rs7853346 CG/GG genotypes was more pronounced in older subjects (age ≥ 60 years), indicating that protective effects of PTENP1 variants may be regulated by specific epidemiological features [24]. Furthermore, we found that the subjects, including nonsmokers and nondrinkers, carrying rs7853346 variant genotype, had a remarkably reduced susceptibility of GC. Some previous studies have demonstrated that tobacco smoking and alcohol drinking were independent risk factors of GC [25, 26]. A possible explanation was that the relation of rs7853346 and GC risk may be dissimulated by accumulated exposure to tobacco carcinogens in smokers or alcohol consumption in drinkers, so that the association in nonsmokers or nondrinkers was more evident [27]. It is important to note that the main reason should be the limited sample size in smokers or drinkers subgroups, which did not have sufficient statistical power to let us find this association. Analogously, in non-FH group, rs7853346 variant genotype could decrease the susceptibility to gastric cancer, but not in FH group, and the explanation should be similar to the smokers or drinkers subgroup. The above results suggested that the occurrence of GC was a complex and multistep process including various genetic and environmental factors. However, further studies are needed to confirm these results. We further found that rs7853346 CG/GG genotypes had a decreased risk of GC among patients with tumor well + moderate differentiation, indicating that different GC differentiated grade regulated by genetic variants could lead to diverse risk of GC.

How the polymorphism rs7853346 in PTENP1 could influence its function was yet unclear. Considering that a single base conversion may change the RNA local secondary structure, we used the RNAfold (http://rna.tbi.univie.ac.at/) [28] algorithm to predict the folding structure of PTENP1 attributed to rs7853346. As a result, rs7853346 may change the folding architectures, revealing that SNPs could involve in the gastric cancer risk through modifying the specific structural motifs of PTENP1 and impacting PTENP1 stability or interaction with other molecules [29, 30]. However, the above results were just our inferences, it is necessary for us to test their authenticity in future researches. In addition, increasing evidences have verified that some lncRNAs functioned as competing endogenous RNAs (ceRNA) decoying miRNAs, which in turn modulated the expression levels of miRNA target genes [31, 32]. Based on the fact that rs7853346 C>G is located in 3′UTR of PTENP1 gene, we speculated that the genetic variant might change target miRNAs. As predicted by RegRNA2.0 (http://regrna2.mbc.nctu.edu.tw/detection.html) [33, 34], the substitution of rs7853346 C>G of PTENP1 may create two microRNA-binding sites on PTENP1, potentially impacting the expression and function of PTENP1. Nevertheless, further studies are required to clarify the precise biological mechanisms of PTENP1 SNPs affecting the expression and function of PTENP1.

Several limitations should be considered. Firstly, the sample size is relatively small, especially for stratified analyses (such as the groups of smokers, drinkers, or FH), limiting the statistical power. We will expand the quantity of sample to make statistical power satisfy stricter criterion in future studies. And when evaluating the influences rs7853346 on PTENP1 expression, the sample size of GG group was relatively small, which could not guarantee enough statistical power. Secondly, some controls recruited from a single hospital during the same period suffered from various nonmalignant diseases, which may cause selection bias. However, the distribution of genotype among controls met the Hardy-Weinberg equilibrium. Also, in the case group, GC patients with poor differentiation accounted for 78%. After taking the well + moderate differentiation group as a sensitivity analysis, we did not find the relationship of rs7853346 with GC risk. The data suggested that the marginally significant results in this study might attribute to this selection bias or residual confounding (e.g., uncontrolled prognosis factors). Thirdly, given the ethical issues, we did not carry out a Helicobacter pylori test for each subject, particularly in controls. Fourthly, we failed to perform the further analyses because of missing relevant clinical data, such as a high-salt diet, chronic gastric ulcer, and BMI, which are also risk factors in gastric carcinogenesis. Finally, our study was conducted in a Chinese population, so the data should be extrapolated to other ethnic groups with caution. Our study also had its own advantages. Firstly, the selection of tagSNPs in PTENP1 was based on the same analysis results of 1000 Genomes Project and Genome Variation Server 144. Secondarily, about analytic strategy, stratified analyses were used to eliminate influences of the potential confounders on GC risk, and Bonferroni's multiple adjustment was conducted to decrease the type I error. Thirdly, the bioinformatics analyses in the present study were therefore more reliable and convictive in the aspect of understanding the potential function of rs7853346 in PTENP1.

## 5. Conclusions

Taken together, the study firstly identified that a potentially functional lncRNA PTENP1 tagSNP (rs7853346 C>G) reduced the susceptibility to GC in a Chinese population. However, further prospective studies with larger sample size are needed to validate the initial findings. In addition, in view of the fact that the difference of PTENP1 expression levels caused by different genotypes was much smaller than that between GC tissues and adjacent normal tissues, so more functional researches should be carried out to illuminate whether rs7853346 decreases gastric cancer risk by altering the expression levels of lncRNA PTENP1 or other specific mechanism.

## Supplementary Material

Supplementary Figture S1. Prediction of the effects of the rs7853346 C>G change on PTENP1 folding structure. The folding structure alterations (a, b) were demonstrated by RNAfold (http://rna.tbi.univie.ac.at/). Supplementary Table S1: The detailed sequences of primers and probes for tag SNPs. Supplementary Table S2: Demographic information. Supplementary Table S3: Association between PTENP1 gene polymorphisms and risk of gastric cancer (rs865005 and rs10971638). Supplementary Table S4: Association between PTENP1 gene polymorphisms and risk of gastric cancer based on the well+moderate differentiation group (rs7853346).

## Figures and Tables

**Table 1:** Demographic information.

Characteristics	Cases (*n* = 768)	Controls (*n* = 768)	*P*
Age (years, mean ± SD)	60.5 ± 9.4	58.6 ± 15.5	0.067
Gender (*n* (%))			
Female	287 (37.4)	319 (41.5)	
Male	481 (62.6)	449 (58.5)	0.095
Hypertension (*n* (%))			
No	526 (68.5)	508 (66.1)	
Yes	242 (31.5)	260 (33.9)	0.327
Diabetes (*n* (%))			
No	686 (89.3)	667 (86.8)	
Yes	82 (10.7)	101 (13.2)	0.135
Smoking status (*n* (%))			
No	556 (72.4)	625 (81.4)	
Yes	212 (27.6)	143 (18.6)	**<0.001**
Drinking status (*n* (%))			
No	593 (77.2)	669 (87.1)	
Yes	175 (22.8)	99 (12.9)	**<0.001**
Residence (*n* (%))			
Rural	440 (57.3)	414 (53.9)	
Urban	328 (42.7)	354 (46.1)	0.182
FH (*n* (%))			
No	703 (91.5)	715 (93.1)	
Yes	65 (8.5)	53 (6.9)	0.250
Tumor differentiation (*n* (%))			
Well + moderate	169 (22)		
Poor	599 (78.0)		
Depth of tumor infiltration (*n* (%))			
T1	163 (21.2)		
T2	97 (12.6)		
T3	323 (42.1)		
T4	185 (24.1)		
Lymph node metastasis (*n* (%))			
Negative	275 (35.8)		
Positive	493 (64.2)		
Localization (*n* (%))			
Cardia	375 (48.8)		
Noncardia	393 (51.2)		

SD, standard deviation; FH, family history of GC. The significant results are in bold.

**Table 2:** Association between PTENP1 gene polymorphisms and risk of gastric cancer (rs7853346).

Genotype	Cases *N* (%)	Controls *N* (%)	Crude OR (95% CI)	*P*	Adjusted OR (95% CI)^∗^	*P*	*P* ^c^
Overall	768	768					
Additive model			**0.79 (0.67–0.94)**	**0.007**	**0.80 (0.67–0.95)**	**0.011**	**0.033**
Codominant model							
CC	496 (64.6)	447 (58.2)	1		1		
CG	241 (31.4)	277 (36.1)	**0.78 (0.63–0.97)**	**0.026**	**0.79 (0.63–0.98)**	**0.033**	0.099
GG	31 (4.0)	44 (5.7)	0.63 (0.39–1.02)	0.060	0.65 (0.40–1.06)	0.087	0.261
Dominant model							
CC	496 (64.6)	447 (58.2)	1		1		
CG+GG	272 (35.4)	321 (41.8)	**0.76 (0.62–0.94)**	**0.010**	**0.77 (0.62–0.95)**	**0.015**	**0.045**
*P* trend						**0.013**	**0.039**
Recessive model							
CC+CG	737 (96.0)	724 (94.3)	1				
GG	31 (4.0)	44 (5.7)	0.69 (0.43–1.11)	0.124	0.71 (0.44–1.14)	0.153	0.459
Allele							
C	1233 (79.8)	1171 (76.2)	1				
G	303 (20.2)	365 (23.8)	**0.79 (0.66–0.94)**	**0.007**			
HWE		0.900					

Abbreviations: OR, odds ratio; CI, confidence interval; HWE, Hardy-Weinberg expectations. ^∗^Adjusted for age, sex, smoking status, drinking status, residence, hypertension, diabetes, and family history of GC in the logistic regression model. *P*^c^ after Bonferroni's correction. *P* trend for CC, CG, GG, and CG+GG genotypes. The significant results are in bold.

**Table 3:** Stratified analyses for PTENP1 genotypes in cases and controls (rs7853346).

Variable	*n* CG+GG (%)/*n* CC (%) for rs7853346		Allelic odds ratios and 95% confidence intervals for rs7853346			
	Cases	Controls	Adjusted OR (95% CI)^∗^	*P* value	*I* ^2^	Heterogeneity *P*
Age (y)						
≥60	153 (19.9)/290 (37.8)	165 (21.5)/226 (29.4)	**0.72 (0.54–0.96)**	**0.026**		
<60	119 (15.5)/206 (26.8)	156 (20.3)/221 (28.8)	0.82 (0.60–1.12)	0.203	0%	0.610
Sex						
Females	107 (13.9)/180 (23.4)	143 (18.6)/176 (22.9)	0.72 (0.51–1.00)	0.052		
Males	165 (21.5)/316 (41.1)	178 (23.2)/271 (35.3)	0.79 (0.60–1.03)	0.085	0%	0.710
Smoking status						
Yes	71 (9.2)/141 (18.4)	57 (7.4)/86 (11.2)	0.76 (0.48–1.22)	0.254		
No	201 (26.2)/355 (46.2)	264 (34.4)/361 (47.0)	**0.78 (0.61–0.99)**	**0.037**	0%	0.863
Drinking status						
Yes	58 (7.6)/117 (15.2)	36 (4.7)/63 (8.2)	0.87 (0.50–1.49)	0.603		
No	214 (27.9)/379 (49.3)	285 (37.1)/384 (50.0)	**0.75 (0.60–0.95)**	**0.016**	0%	0.718
Residence						
Rural	166 (21.6)/274 (35.7)	186 (24.2)/228 (29.7)	0.77 (0.59–1.02)	0.071		
Urban	106 (13.8)/222 (28.9)	135 (17.6)/219 (28.5)	0.78 (0.57–1.08)	0.133	0%	0.960
FH						
Yes	20 (2.6)/45 (5.9)	20 (2.6)/33 (4.3)	0.69 (0.31–1.55)	0.371		
No	252 (32.8)/451 (58.7)	301 (39.2)/414 (53.9)	**0.78 (0.62–0.97)**	**0.023**	0%	0.841

OR, odds ratio; CI, confidence interval; FH, family history of GC. ^∗^Adjusted for age, sex, smoking status, drinking status, residence, hypertension, diabetes, and family history of GC (excluding the stratified factor in each stratum) in the logistic regression model. The significant results are in bold.

**Table 4:** Associations between variant PTENP1 genotypes and clinicopathologic characteristics of gastric cancer (rs7853346).

Variable	CG+GG, CC for rs7853346		Allelic odds ratios and 95% confidence intervals for rs7853346	
	CG+GG, *n*	CC, *n*	Adjusted OR (95% CI)^∗^	*P* value
Tumor differentiation				
Well + moderate	70	99		
Poor	202	397	**0.70 (0.49–1.00)**	**0.048**
Depth of tumor infiltration				
T1	64	99	1	
T2	34	63	0.79 (0.46–1.35)	0.383
T3	101	222	0.69 (0.46–1.03)	0.071
T4	73	112	1.01 (0.64–1.59)	0.963
*P* trend				0.716
Lymph node metastasis				
Negative	107	168		
Positive	165	328	0.77 (0.57–1.05)	0.097
Localization				
Cardia	131	244		
Noncardia	141	252	1.05 (0.78–1.42)	0.761

OR, odds ratio; CI, confidence interval. ^∗^Adjusted for age, sex, smoking status, drinking status, residence, hypertension, diabetes, and family history of GC in the logistic regression model. The significant results are in bold.
