# Supplementary figures and images for: Polymorphisms in lncRNA PTENP1 and the Risk of Gastric Cancer in a Chinese Population
Source: Dis Markers. 2017 Aug 28;2017:6807452. doi: 10.1155/2017/6807452 (PMC5592395; doi:10.1155/2017/6807452)

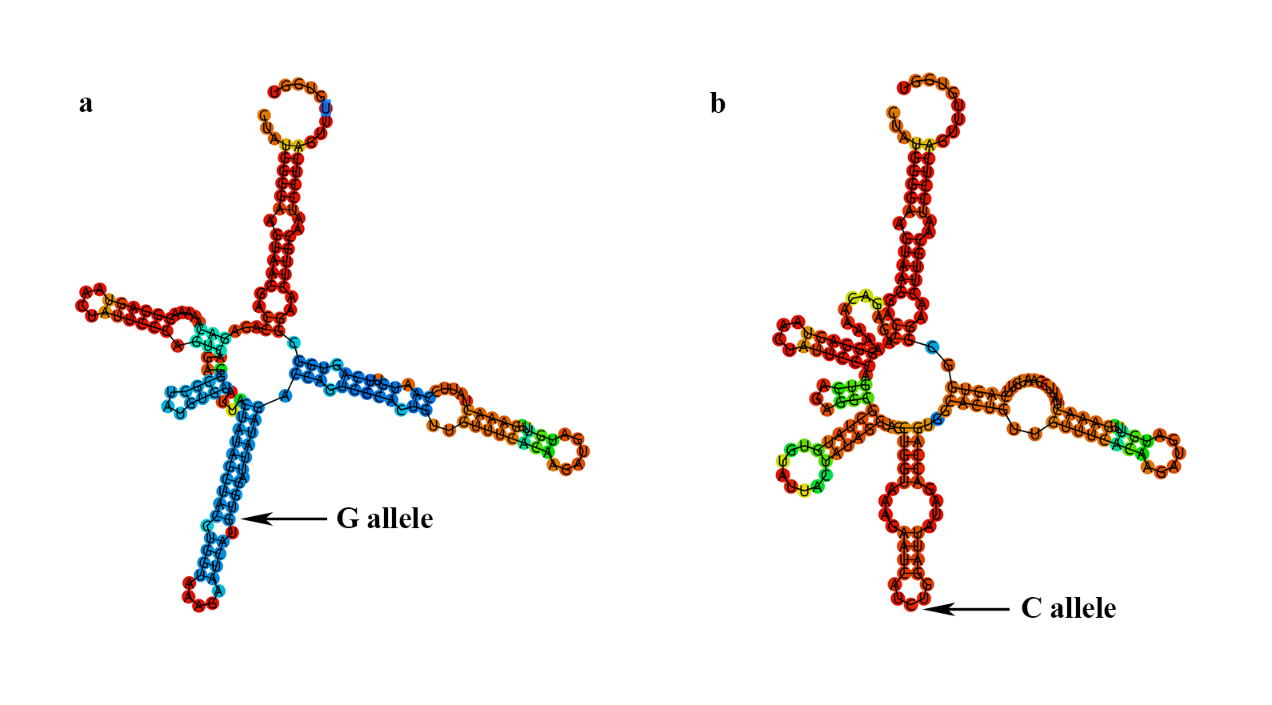

Supplement: Supplementary file 2 [file 6807452.f2.docx]
