# Supplementary material for: Polymorphisms in lncRNA PTENP1 and the Risk of Gastric Cancer in a Chinese Population
Source: Dis Markers. 2017 Aug 28;2017:6807452. doi: 10.1155/2017/6807452 (PMC5592395; doi:10.1155/2017/6807452)
Supplement: Supplementary file 3 [file 6807452.f3.docx]

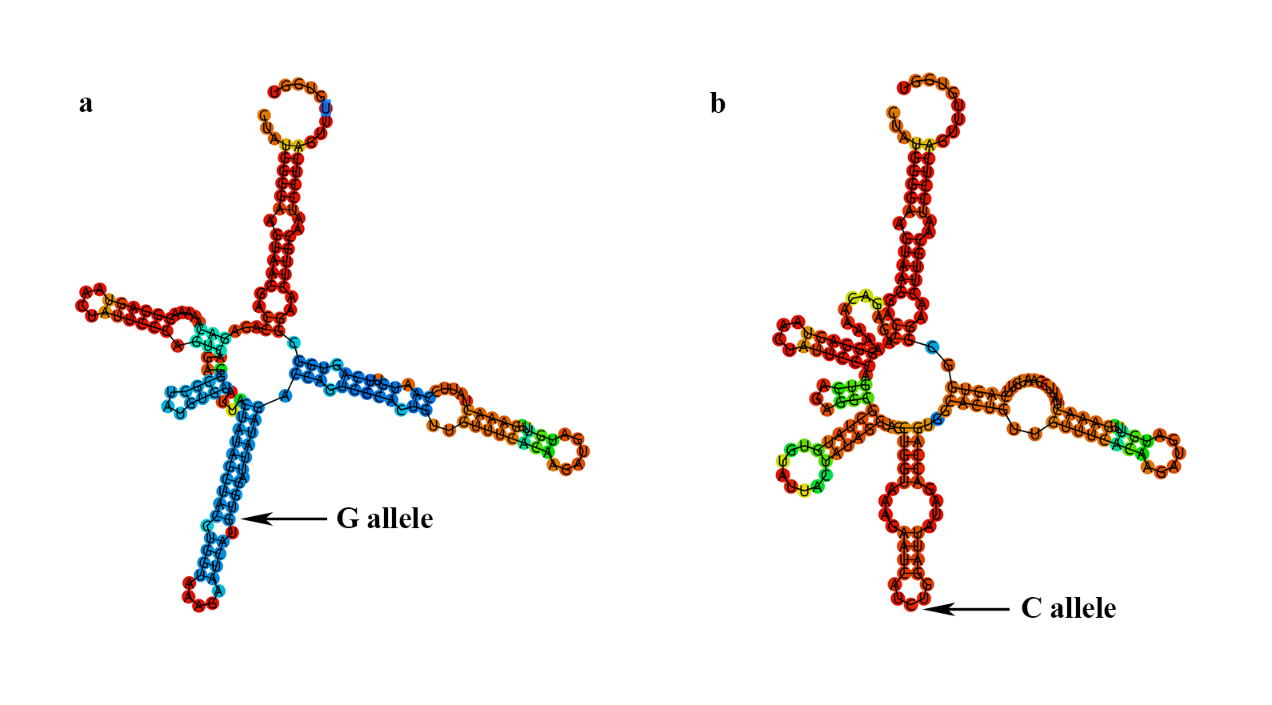


**Supplementary Figture S1. Prediction of the effects of the rs7853346 C>G change on PTENP1 folding structure.** The folding structure alterations (a, b) were demonstrated by RNAfold (<http://rna.tbi.univie.ac.at/>).

**Supplementary Table S1:** The detailed sequences of primers and probes for tag SNPs

| **SNPs** | **Primer sequence (5’-3’)** | **Probe sequence** |
| --- | --- | --- |
| rs7853346 | F-CCACAAACTGAGGATTGCAAGTT | C: FAM-TCTATAATCCACATGATTCT-MGB |
| C>G | R-CAGTCAGAGGCGCTATGTGTATTAC | G: HEX- TCTATAATCCAGATGATTC-MGB |
| rs865005 | F-TCGCCTGTCACCATTTCCA | C: FAM-TTGGTCTCTCCCCTTC-MGB |
| C>T | R-CGCCGTGTTGGAGGCA | T: HEX-TTGGTCTTTCCCCTTC-MGB |
| rs10971638 | F-GGAATGAACCTTCTGCAACATCT | A: FAM-TCCACAAATGAAGGAATA-MGB |
| G>A | R-TTGTTGCTGTGTTTCTTATCTATGACA | G: HEX-CCACAAATGAAGGGAT-MGB |

**Supplementary Table S2:** Demographic information

| **Characteristics** | **Cases (n = 768)** | **NRP (n = 87)** | ***P*** |
| --- | --- | --- | --- |
| Age(years, mean±SD) | 60.5±9.4 | 61.4±8.9 | 0.407 |
| Gender, (n (%)) |  |  |  |
| Female | 287 (37.4) | 37 (42.5) |  |
| Male | 481 (62.6) | 50 (57.5) | 0.347 |
| Hypertension, (n (%)) |  |  |  |
| No | 526 (68.5) | 57 (65.5) |  |
| Yes | 242 (31.5) | 30 (34.5) | 0.573 |
| Diabetes, (n (%)) |  |  |  |
| No | 686 (89.3) | 72 (82.8) |  |
| Yes | 82 (10.7) | 15 (17.2) | 0.067 |
| Smoking status, (n (%)) |  |  |  |
| No | 556 (72.4) | 60 (69.0) |  |
| Yes | 212 (27.6) | 27 (27.0) | 0.499 |
| Drinking status, (n (%)) |  |  |  |
| No | 593 (77.2) | 65 (74.7) |  |
| Yes | 175 (22.8) | 22 (25.3) | 0.600 |
| Residence, (n (%)) |  |  |  |
| Rural | 440 (57.3) | 61 (70.1) |  |
| Urban | 328 (42.7) | 26 (29.9) | **0.021** |
| FH, (n (%)) |  |  |  |
| No | 703 (91.5) | 82 (94.3) |  |
| Yes | 65 (8.5) | 5 (5.7) | 0.381 |
| Tumor differentiation (n (%)) |  |  |  |
| Well + Moderate | 169 (22) | 16 (18.4) |  |
| Poor | 599 (78.0) | 71 (81.6) | 0.438 |
| Depth of tumor infiltration (n(%)) |  |  |  |
| T1 | 163 (21.2) | 24 (27.6) |  |
| T2 | 97 (12.6) | 8 (9.2) |  |
| T3 | 323 (42.1) | 31 (35.6) |  |
| T4 | 185 (24.1) | 24 (27.6) | 0.338 |
| Lymph node metastasis (n (%)) |  |  |  |
| Negative | 275 (35.8) | 29 (33.3) |  |
| Positive | 493 (64.2) | 58 (66.7) | 0.648 |
| Localization (n (%)) |  |  |  |
| Cardia | 375 (48.8) | 46 (52.9) |  |
| Noncardia | 393 (51.2) | 41 (47.1) | 0.474 |

Abbreviations: NRP, non-response patients; SD, standard deviation; FH, family history of GC

The significant results are in bold.

**Supplementary Table S3:** Association between PTENP1 gene polymorphisms and risk of gastric cancer (rs865005 and rs10971638)

| **Genotype** | **Cases N (%)** | **Controls N (%)** | **Crude OR**  **(95% CI)** | ***P*** | **Adjusted OR**  **(95% CI)*** | ***P*** | ***P*^c^** |
| --- | --- | --- | --- | --- | --- | --- | --- |
| overall | 768 | 768 |  |  |  |  |  |
| rs865005 |  |  |  |  |  |  |  |
| Additive model |  |  | 1.07 (0.84-1.34) | 0.594 | 1.08 (0.85-1.37) | 0.517 | 1.000 |
| Co-dominant model |  |  |  |  |  |  |  |
| CC | 622 (81.0) | 632 (82.3) | 1 |  | 1 |  |  |
| CT | 136 (17.7) | 125 (16.3) | 1.11 (0.85-1.44) | 0.461 | 1.13 (0.86-1.49) | 0.372 | 1.000 |
| TT | 10 (1.3) | 11 (1.4) | 0.92 (0.39-2.19) | 0.857 | 0.88 (0.36-2.13) | 0.775 | 1.000 |
| Dominant model |  |  |  |  |  |  |  |
| CC | 622 (81.0) | 632 (82.3) | 1 |  | 1 |  |  |
| CT+ TT | 146 (19.0) | 136 (17.7) | 1.09 (0.84-1.41) | 0.510 | 1.12 (0.86-1.45) | 0.416 | 1.000 |
| *P* trend |  |  |  |  |  | 0.355 | 1.000 |
| Recessive model |  |  |  |  |  |  |  |
| CC + CT | 758 (98.7) | 757 (98.6) | 1 |  | 1 |  |  |
| TT | 10 (1.3) | 11 (1.4) | 0.91 (0.38-2.15) | 0.826 | 0.87 (0.36-2.10) | 0.758 | 1.000 |
| Allele |  |  |  |  |  |  |  |
| C | 1380 (89.8) | 1389 (90.4) | 1 |  |  |  |  |
| T | 156 (10.2) | 147 (9.6) | 1.07 (0.84-1.35) | 0.586 |  |  |  |
| HWE |  | 0.098 |  |  |  |  |  |
| rs10971638 |  |  |  |  |  |  |  |
| Additive model |  |  | 1.02 (0.82-1.28) | 0.863 | 1.06 (0.84-1.33) | 0.637 | 1.000 |
| Co-dominant model |  |  |  |  |  |  |  |
| GG | 602 (78.4) | 610 (79.4) | 1 |  | 1 |  |  |
| GA | 159 (20.7) | 146 (19.0) | 1.10 (0.86-1.42) | 0.442 | 1.14 (0.88-1.47) | 0.311 | 0.933 |
| AA | 7 (0.9) | 12 (1.6) | 0.59 (0.23-1.51) | 0.267 | 0.62 (0.24-1.63) | 0.334 | 1.000 |
| Dominant model |  |  |  |  |  |  |  |
| GG | 602 (78.4) | 610 (79.4) | 1 |  | 1 |  |  |
| GA+ AA | 166 (21.6) | 158 (20.6) | 1.07 (0.83-1.36) | 0.617 | 1.10 (0.86-1.42) | 0.434 | 1.000 |
| *P* trend |  |  |  |  |  | 0.441 | 1.000 |
| Recessive model |  |  |  |  |  |  |  |
| GG + GA | 761 (99.1) | 756 (98.4) | 1 |  | 1 |  |  |
| AA | 7 (0.9) | 12 (1.6) | 0.58 (0.23-1.48) | 0.248 | 0.62 (0.24-1.61) | 0.323 | 0.969 |
| Allele |  |  |  |  |  |  |  |
| G | 1363 (88.7) | 1366 (88.9) | 1 |  |  |  |  |
| A | 173 (11.3) | 170 (11.1) | 0.98 (0.78-1.23) | 0.864 |  |  |  |
| HWE |  | 0.342 |  |  |  |  |  |

Abbrevations: OR, odds ratio; CI, confidence interval; HWE, Hardy–Weinberg expectations.

*Adjusted for age, sex, smoking status, drinking status, residence, hypertension, and diabetes, Family history of GC in logistic regression model.

*P* trend for CC, CG, GG, and CG+GG genotypes.

*P*^c^ after Bonferroni correction.

**Supplementary Table S4:** Association between PTENP1 gene polymorphisms and risk of gastric cancer based on the well+moderate differentiation group (rs7853346)

| **Genotype** | **Cases N (%)** | **Controls N (%)** | **Crude OR**  **(95% CI)** | ***P*** | **Adjusted OR**  **(95% CI)*** | ***P*** | ***P*^c^** |
| --- | --- | --- | --- | --- | --- | --- | --- |
| overall | 169 | 768 |  |  |  |  |  |
| Additive model |  |  | 1.00 (0.76-1.31) | 0.971 | 1.02 (0.77-1.35) | 0.918 | 1.000 |
| Co-dominant model |  |  |  |  |  |  |  |
| CC | 99 (58.6) | 447 (58.2) | 1 |  | 1 |  |  |
| CG | 60 (35.5) | 277 (36.1) | 0.98 (0.69-1.39) | 0.902 | 0.98 (0.69-1.41) | 0.916 | 1.000 |
| GG | 10 (5.9) | 44 (5.7) | 1.03 (0.50-2.11) | 0.944 | 1.08 (0.51-2.26) | 0.848 | 1.000 |
| Dominant model |  |  |  |  |  |  |  |
| CC | 99 (58.6) | 447 (58.2) | 1 |  | 1 |  |  |
| CG+ GG | 70 (41.4) | 321 (41.8) | 0.99 (0.70-1.38) | 0.928 | 1.00 (0.71-1.41) | 0.986 | 1.000 |
| *P* trend |  |  |  |  |  | 0.979 | 1.000 |
| Recessive model |  |  |  |  |  |  |  |
| CC + CG | 159 (94.1) | 724 (94.3) | 1 |  |  |  |  |
| GG | 10 (5.9) | 44 (5.7) | 1.04 (0.51-2.10) | 0.924 | 1.12 (0.55-2.30) | 0.756 | 1.000 |
| Allele |  |  |  |  |  |  |  |
| C | 258 (76.3) | 1171 (76.2) | 1 |  |  |  |  |
| G | 80 (23.7) | 365 (23.8) | 1.00 (0.75-1.31) | 0.971 |  |  |  |
| HWE |  | 0.900 |  |  |  |  |  |

Abbreviations: OR, odds ratio; CI, confidence interval; HWE, Hardy–Weinberg expectations.

*Adjusted for age, sex, smoking status, drinking status, residence, hypertension, and diabetes, Family history of GC in logistic regression model.

*P*^c^ after Bonferroni correction.

*P* trend for CC, CG, GG, and CG+GG genotypes.
